# Assessment of quality and readability of information provided by ChatGPT in relation to developmental dysplasia of the hip and periacetabular osteotomy

**DOI:** 10.1093/jhps/hnaf025

**Published:** 2025-05-08

**Authors:** Vincent J Leopold, Stephen Fahy, Carsten Perka, Jens Goronzy, George Grammatopoulos, Paul E Beaulé, Sebastian Hardt

**Affiliations:** Center for Musculoskeletal Surgery, Charité—Universitätsmedizin Berlin, Chariteplatz 1, Berlin 10117, Germany; Center for Musculoskeletal Surgery, Charité—Universitätsmedizin Berlin, Chariteplatz 1, Berlin 10117, Germany; Center for Musculoskeletal Surgery, Charité—Universitätsmedizin Berlin, Chariteplatz 1, Berlin 10117, Germany; University Center for Orthopaedics, Trauma and Plastic Surgery, University Hospital Carl Gustav Carus, Fetscherstraße 74, Dresden 10117, Germany; Division of Orthopaedic Surgery, University of Ottawa, The Ottawa Hospital, 501 Smyth Rd, Ottawa, Ontario K1H 8L6, Canada; Division of Orthopaedic Surgery, University of Ottawa, The Ottawa Hospital, 501 Smyth Rd, Ottawa, Ontario K1H 8L6, Canada; Center for Musculoskeletal Surgery, Charité—Universitätsmedizin Berlin, Chariteplatz 1, Berlin 10117, Germany

## Abstract

This study evaluates the quality and readability of responses given by ChatGPT 4 relating to common patient queries on Developmental Dysplasia of the Hip (DDH) and Periacetabular Osteotomy (PAO). Frequently asked questions on DDH and PAO were selected from online Patient Education Materials and posed to ChatGPT 4. The responses were evaluated by four high-volume PAO surgeons using a well-established evidence-based rating system, categorizing responses from ‘excellent response not requiring clarification’ to ‘unsatisfactory requiring substantial clarification’. Readability assessments were subsequently conducted to determine the required literacy level to understand the content provided. Responses from ChatGPT 4 varied significantly between preoperative and postoperative queries. In the postoperative category, 50% of responses were rated as ‘excellent’, showing no need for further clarification, while the preoperative responses frequently required minimal to moderate clarification. The overall median response rating was ‘satisfactory requiring minimal clarification’. Readability tests showed that the average Reading Grade Level was 13.44, considerably higher than the recommended sixth-grade level for patient education materials, indicating a substantial barrier to comprehension for the general public. While ChatGPT delivers generally reliable information, the complexity of its language is a major barrier to widespread utilization as a tool for patient education. Future iterations of ChatGPT should aim to utilize more simplistic language, as such enhancing accessibility without compromising content quality.

## Introduction

The advent of Artificial Intelligence (AI) in healthcare has opened new avenues for patient education and interaction [1–4]. While Large Language Models, such as ChatGPT, have shown promise as a tool for patient education in total hip arthroplasty, their role in the context of hip-preservation surgery remains largely unexplored [5]. Periacetabular Osteotomy (PAO) is recognized as a highly effective treatment option for Developmental Dysplasia of the Hip (DDH), particularly in young adults. Clinical studies have consistently reported excellent outcomes, with significant improvements in hip function and pain relief [6–12]. These positive results underscore PAO’s role as a cornerstone in the management of DDH, aiming to preserve the native hip joint and delay or prevent the progression to osteoarthritis [13–16].

Patients frequently cite the Internet as a valuable resource for education, reporting that internet-based resources are equivalent to, or better than, information received from healthcare providers [17, 18]. DDH predominantly affects younger individuals who are inherently more engaged with digital media [14, 19–22]. This demographic’s propensity for digital information consumption highlights the need for accurate online medical resources [20, 21]. While the accuracy of Patient Education Materials is of paramount importance, the readability of these documents is a vital component in improving health literacy. The average American reads at an eighth-grade reading level, and as such, Patient Education Materials must be written at this level to optimize utility [23, 24]. Despite this Patient Education Materials and Internet-based resources are consistently written at levels far exceeding the average level of literacy of the general public [25].

The aim of our study was to evaluate the potential of large language models like ChatGPT4 in educating patients about DDH and PAO. The evaluation of ChatGPT 4’s responses to common patient questions in this domain is an essential step in determining the feasibility of using generative AI as a tool for patient education in the field of orthopaedics. By exploring the utility of ChatGPT in this specialized field, this study seeks to contribute to the understanding of the role of generative AI tools in patient education, specifically in hip-preservation surgery. The aim of this study was to assess whether ChatGPT 4 can provide patients with rapid access to accurate and accessible information about their conditions and treatment options. We hypothesized that the responses provided by ChatGPT would align with current literature and expert opinions in the field of hip-preservation surgery and that ChatGPT would provide answers that are readable and understandable to the patient.

## Materials and methods

### Selection of questions

On 10 January 2024 the terms ‘Periacetabular Osteotomy’, ‘Developmental Dysplasia of the Hip’, and ‘Hip Osteotomy’ were entered into a freshly installed Google (www.google.com) Internet browser. The results yielded were reviewed by the main Author (VL), identifying Hospital-based Patient Education Materials. The (FAQs) posed within these Patient Education Materials were saved in a Microsoft Word Document. A list of 26 questions regarding DDH and PAO was initially created. This curated list was then subsequently reviewed by two authors specialized in hip preservation surgery, and 22 questions were selected for analysis (see Appendix). The questions were divided into questions relating to the preoperative (questions 1–7) and postoperative period (questions 8–22).

### ChatGPT interaction

The selected questions were posed to ChatGPT 4 in a conversational format with use of ChatGPT 4 (https://chat.openai.com/) on 12 January 2024. Responses were recorded after the initial query, with no follow-up or repeated queries. The responses were saved in separate Microsoft Word documents for subsequent analysis.

### Response evaluation

ChatGPT 4’s responses were rated by four high-volume PAO surgeons using a previously established rating system [26]. The answers were categorized as 1: ‘excellent response not requiring clarification’, 2: ‘satisfactory requiring minimal clarification’, 3: ‘satisfactory requiring moderate clarification’, and 4: ‘unsatisfactory requiring substantial clarification’ [26]. The evaluating surgeons were experts in hip preservation surgery, with at least 5 years of experience. Each surgeon has performed a high mid-three-digit number of PAO procedures at academic orthopaedic centres.

### Readability

To assess the readability of both the questions asked and responses provided the Readability Studio Professional Edition Program (Oleander Software Ltd, Version 2019) was used. This software assesses the readability of documents using six frequently used assessment tools: The Flesch–Kincaid Reading Grade Level, the SMOG score, the Fry Score, the Gunning Fog Score, the Raygor Estimate, and the Flesch Reading Ease Index (FRES) (Appendix 2). The Reading Grade Level is an estimation of the level of education and literacy required to read, understand, and retain the information contained within an article. The reading grade levels were reported as the standard US grade level. The average reading grade level of Americans is at the eighth grade (ages 13–14). Consequently, experts advise that Patient Education Materials be composed at a sixth-grade level (ages 11–12) to enhance their readability [23, 24, 27, 28]. The FRES Index expresses readability as an index score ranging from 0 to 100, higher scores indicate superior readability.

### Statistical analysis

Descriptive Statistics: To evaluate the rating of the responses given by ChatGPT 4 mode, median, and percentage distribution of ratings for each question were calculated. The percentage distribution illustrates the proportion of each rating from 1 to 4. Kendall’s W (Coefficient of Concordance) was used to assess inter-rater reliability [29]. For documentation of the collected data, Microsoft Excel (Microsoft Corp. Released 2016. Excel Professional for Windows, Version 16.16.2. Redmond, WA: Microsoft Corp.) was used. The collected data were analysed using IBM SPSS 25 (IBM Corp. Released 2017. IBM SPSS Statistics for Windows, Version 25.0. Armonk, NY: IBM Corp.).

## Results

### Response evaluation

The overall evaluation of ChatGPT 4’s responses across all questions revealed the following descriptive statistics: The mode rating was 2, indicating that responses were generally perceived as ‘satisfactory, requiring minimal clarification’. The median rating was also 2, suggesting that the typical response fell into this category. The percentage distribution of ratings showed that 37% of the responses were rated as ‘excellent’ (rating 1), 35% as ‘satisfactory, requiring minimal clarification’ (rating 2), 18% as ‘satisfactory, requiring moderate clarification’ (rating 3), and 10% as ‘unsatisfactory’ (rating 4). A detailed overview of ratings is provided in Table 1.

**Table 1.** Descriptive statistics of ChatGPT responses: Questions 1–7 refer to the preoperative period, while Questions 8–22 refer to the postoperative period. ‘Mode’ indicates the most frequently occurring rating for each question, while ‘Median’ represents the middle value separating the higher.

Question	Modus	Median	1 (%)	2 (%)	3 (%)	4 (%)
1. What is hip dysplasia?	2	2.0	0	100	0	0
2. How is hip dysplasia diagnosed?	2	2.0	25	50	25	0
3. What are the symptoms of hip dysplasia (DDH)?	2	2.0	25	50	25	0
4. What happens to the hip in hip dysplasia?	1	1.5	50	25	25	0
5. What are the treatment options for hip dysplasia?	2	2.5	0	50	50	0
6. What is done in periacetabular osteotomy?	1	2.5	25	25	25	25
7. Do I need to stop smoking before PAO?	1	1.0	75	25	0	0
8. When can I walk after PAO?	1	1.5	50	50	0	0
9. What are the possible complications of PAO?	2	2.0	0	75	0	25
10. When can I work after PAO?	1	2.0	50	0	50	0
11. When can I play sports again after PAO?	1	1.5	50	25	0	25
12. How long will it take to heal after PAO?	1	1.0	75	25	0	0
13. How long does the surgery take?	1	1.5	50	50	0	0
14. How long is the hospital stay after PAO?	1	1.0	100	0	0	0
15. Will I need to wear a brace after PAO?	1	2.5	50	0	0	50
16. Will I need to have my screw removed after PAO?	1	1.5	50	50	0	0
17. Does the labrum also need to be repaired in PAO?	3	3.0	25	0	75	0
18. Will I need hip replacement after PAO?	1	1.0	75	25	0	0
19. When can I drive after PAO?	1	1.5	50	25	25	0
20. Why do I have to take blood thinners after PAO?	1	1.0	75	25	0	0
21. When can I have sex after PAO?	1	1.5	50	25	25	0
22. How often do I need to see the doctor after PAO and will I get x-rays?	1	2.0	50	0	50	0

### Preoperative period (Questions 1–7)

The evaluation of ChatGPT 4’s responses for the preoperative period (Questions 1–7) showed that the mode rating was 2, indicating responses were generally perceived as ‘satisfactory, requiring minimal clarification’. The median rating was also 2. The percentage distribution revealed 25% of responses were rated as ‘excellent’ (rating 1), 40% as ‘satisfactory, requiring minimal clarification’ (rating 2), 25% as ‘satisfactory, requiring moderate clarification’ (rating 3), and 10% as ‘unsatisfactory’ (rating 4).

### Postoperative period (Questions 8–22)

For the postoperative period (Questions 8–22), the mode rating was 1, suggesting many responses were viewed as ‘excellent responses not requiring clarification’. The median rating was also 1. The percentage distribution showed 50% of responses were rated as ‘excellent’ (rating 1), 30% as ‘satisfactory, requiring minimal clarification’ (rating 2), 15% as ‘satisfactory, requiring moderate clarification’ (rating 3), and 5% as ‘unsatisfactory’ (rating 4).

Table 1: Descriptive Statistics of ChatGPT Responses: Questions 1–7 refer to the preoperative period, while Questions 8–22 refer to the postoperative period. ‘Mode’ indicates the most frequently occurring rating for each question, while ‘Median’ represents the middle value separating the higher half from the lower half of the ratings. ‘1 (%)’, ‘2 (%)’, ‘3 (%)’, and ‘4 (%)’ indicate the percentage of responses rated as excellent, satisfactory (minimal clarification), satisfactory (moderate clarification), and unsatisfactory, respectively.

### Inter-rater reliability

The overall Kendall’s W coefficient for inter-rater reliability across all questions was 0.350, indicating a moderate level of agreement among the evaluators. Specifically, for the preoperative questions (1–7), Kendall’s W was 0.300, suggesting moderate agreement among raters for this period. For the postoperative questions (8–22), Kendall’s W was higher at 0.400, indicating better agreement among evaluators for these responses.

### Readability

**Figure 1.**
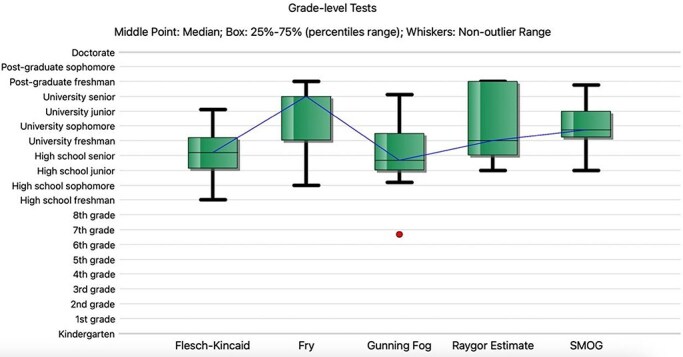
Reading grade level for answers to FAQs provided by ChatGPT 4. The horizontal line represents the median; the top and bottom edges of each box display the interquartile range; whiskers extend to the lower and upper quartiles; circles mark outliers.

The mean reading grade level of the questions posed by the investigators was 9.9 (FORCAST Score, Suggested Reader Age 14–15), slightly above the average eighth-grade reading level of the general American public. The mean reading grade level for answers given by ChatGPT 4.0 was 13.44 (range, 12–15) (Fig. 1). The mean Flesch Reading Ease Score was 32. None of the responses provided by ChatGPT 4 were at or below the average eighth-grade reading grade level of the general American public. The responses given by ChatGPT4 exceeded the recommended eighth-grade reading level by an average of 5.44 reading grade levels. For a detailed overview of Readability assessment see Table 2.

**Table 2.** Readability assessment for responses provided by ChatGPT 4.0.

Test	Valid N	Minimum	Maximum	Range	Mode(s)	Means
Flesch–Kincaid	22	9	15.1	6.1	11; 12; 13	12.1
Fry	22	10	17	7	16	15
Gunning Fog	22	6.7	16.1	9.4	11	12
Raygor Estimate	22	11	17	6	17	14
SMOG	22	11	16.8	5.8	13	14.1
Mean						13.44

## Discussion

This study investigated the quality and readability of responses provided by ChatGPT 4 in relation to questions on DDH and PAO. ChatGPT 4’s responses were generally satisfactory, with a marked difference in performance between the preoperative and postoperative contexts.

Our first hypothesis proposed that ChatGPT 4’s responses would align with the current literature and expert opinions in the field of hip-preservation surgery. This is partially supported by our results. The moderate agreement among evaluators indicates that ChatGPT 4 provides a consistent and reliable level of response. However, there was a clear variation in the quality of responses given between questions relating to the preoperative period when compared with questions relating to the postoperative period. The postoperative period responses were generally more accurate, possibly because these questions tend to be more direct and factual. In contrast, the preoperative questions, which often require complex decision-making and detailed explanations, may be challenging for the large language models’ capability to deliver comprehensive and nuanced information.

The observed heterogeneity in expert ratings highlights the challenges of achieving consensus when evaluating AI-generated responses, particularly for complex or nuanced medical questions. For example, in question #9, addressing possible complications in PAO, three reviewers rated the response as ‘2: Satisfactory requiring minimal clarification’, while one reviewer rated it as ‘4: Unsatisfactory requiring substantial clarification’. This discrepancy could be linked to the lack of differentiation in ChatGPT’s response between common and rare complications, which may have led to varying expectations among reviewers. The single rating of ‘4’ may also reflect the individual clinical experiences of that reviewer. If a specific complication that the reviewer has encountered or considers particularly significant was not adequately highlighted or mentioned, this could have influenced their assessment. In contrast, the other reviewers may have focused more on general accuracy and comprehensiveness, leading to a more favourable evaluation. This example underscores the influence of personal experiences and priorities in expert assessments, highlighting the importance of refining AI-generated responses to balance comprehensiveness with contextual relevance. Additionally, it reinforces the critical role of surgeons in supplementing AI-generated content with tailored, patient-specific explanations to ensure all relevant information is conveyed effectively.

Another example of heterogeneity in expert ratings is seen with question #6, ‘What is done in periacetabular osteotomy’, where the four reviewers assigned ratings ranging from 1 to 4. This variability may be attributed to differences in surgical techniques practised by the reviewers, who represent four surgeons from three centres across two countries. Each centre might utilize slightly distinct approaches or emphasize different aspects of the procedure, such as specific surgical steps, instrumentation, or postoperative protocols. ChatGPT’s response may not fully capture these nuances, leading reviewers to evaluate its quality differently based on how well it aligns with their own practices. For instance, a reviewer whose technique or priorities are underrepresented in the response may perceive it as requiring substantial clarification. Conversely, a response that broadly addresses the standard principles of the procedure might be rated more favourably by another reviewer who values generalizability. This observation underscores the challenge for AI tools to generate content that is both accurate and representative across diverse practices. It also highlights the importance of incorporating multicentre and international perspectives when evaluating AI-generated medical content. Future iterations of AI could benefit from training on more globally representative datasets to better address regional and institutional variations in clinical practice.

While assessing the current capabilities of ChatGPT in providing responses to complex medical queries, it is important to recognize that AI and large language models are continuously evolving technologies. According to previous studies, newer versions of these tools have shown potential for generating significantly better-quality information. This improvement indicates that future versions of AI tools are likely to offer even higher-quality answers, further enhancing their potential role in patient education [1, 30, 31]. Furthermore, newer versions have enhanced their ability to cite sources accurately. In contrast to earlier versions of ChatGPT where no sources were provided, responses now include links to sources, with a significant portion directing to peer-reviewed research [30]. This progression in source citation accuracy marks a significant step forward in using AI for patient education. Accurate citations are crucial as they allow both patients and healthcare providers to verify the scientific validity of the information independently. Healthcare providers and patient organizations must work with developers to ensure future models emphasize the use of high-quality, verifiable sources. Improved citation practices enhance the credibility and reliability of the information provided, empowering physicians to recommend these AI-generated resources with greater confidence. Despite the progress in source citation accuracy, accessibility to high-quality literature remains a significant barrier. Much of the peer-reviewed content referenced by AI tools is behind paywalls, limiting access for patients and healthcare providers. Open access initiatives are crucial to ensure that evidence-based information is available to a broader audience. In addition to accessibility, the complexity of medical information presents another challenge. Even with open access information, patients in most cases would not have the research and health literacy to read, comprehend and critically appraise scientific literature. One possible solution could be the development of chatbots by medical societies, trained with curated medical information provided by field experts. Efforts to enhance both accessibility and readability will ensure that AI tools can effectively bridge gaps in patient education and support informed decision-making.

Our second hypothesis was that ChatGPT 4 would provide answers to patients at an appropriate reading grade level for the general public. Analysis using various readability indices revealed that the mean Reading Grade Level for responses was far higher than the reading ability of the average American, suggesting that they are more suitable for individuals with at least a high school education. The Flesch Reading Ease Score of 32 indicates that while responses are detailed, they are also quite challenging to read. This highlights a significant area for improvement in making AI-generated content more accessible to patients with varying levels of literacy. While large language models can be instructed to simplify their responses using targeted prompts, this often results in content that is easier to understand but may lack detail and accuracy. These nuances, although important, might not be apparent to lay readers, potentially reducing the depth of the conveyed information. This potential loss underscores a critical limitation: the irreplaceable value of direct physician-patient interaction. In these interactions, a physician can tailor explanations to the patient’s specific level of understanding and information needs, an adaptability that AI cannot fully replicate. This nuanced approach is vital in medical settings where understanding and trust are paramount. It underscores the role of AI in patient education as a supplement rather than a substitute.

This study has to be viewed in the light of its limitations. First, the evaluation of ChatGPT’s responses was based primarily on expert opinions focused on the factual accuracy of the answers. This approach lacks a patient perspective, which is crucial for assessing the real-world effectiveness of AI in patient education. Additionally, the readability of the responses was measured using a relatively generic approach. Future studies should incorporate the patient perspective to better understand how AI and physicians can collaboratively meet patient needs for medical information. Furthermore, future studies should implement standardized consensus processes to reduce variability introduced by individual surgeon preferences, which may influence study outcomes. Second, AI technology and large language models in particular are rapidly evolving. The findings presented in this study represent a snapshot based on the current capabilities of large language models. It is anticipated that future versions of these models will become even more sophisticated. Therefore, the applicability of large language models for patient information and education needs continuous evaluation in future research to keep pace with technological advancements. Third, the study was conducted as a multicentre study involving experts from three centres across two countries. This setup might account for some variation in the evaluation of ChatGPT’s responses, as differing practices and perspectives among these centres could influence how questions are assessed. Such variability highlights the challenge of standardizing practices across diverse clinical environments.

## Conclusion

This study evaluated the quality and readability of ChatGPT responses on DDH and PAO. A major challenge identified is the text complexity, which often exceeds the recommended reading grade level for patient education materials, potentially limiting their comprehensibility and public accessibility.

To enhance the utility of large language models like ChatGPT in medical communication, we recommend simplifying the language to meet or fall below the eighth-grade reading level, thus improving readability without sacrificing information depth. Collaborative efforts between developers, healthcare providers, and patient organizations should focus on regular updates and scientific accuracy of the content. Additionally, improving the transparency of source citations will help verify the scientific validity of the information provided.

These strategies will help evolve large language models like ChatGPT into more effective tools for patient education, empowering users with accessible information that supports informed healthcare decisions and enhances digital health literacy.

## Supplementary Material

hnaf025_Supp

## Data Availability

The data underlying this article will be shared upon reasonable request from the corresponding author.
