# Supplementary material for: Assessment of quality and readability of information provided by ChatGPT in relation to developmental dysplasia of the hip and periacetabular osteotomy
Source: J Hip Preserv Surg. 2025 May 8;12(4):242–7. doi: 10.1093/jhps/hnaf025 (PMC12712974; doi:10.1093/jhps/hnaf025)
Supplement: hnaf025_Supp [file hnaf025_supp.zip › suppl_data/Supplemental FAQ+ChatGPT responses.docx]

**Frequently asked questions: PAO in DDH**

**Questions 1-7 refer to the preoperative period**

**Questions 8-22 refer to the postoperative period**

1. **What is hip dysplasia?**

Hip dysplasia is a condition where the hip joint, which is a ball-and-socket joint, does not develop properly in infants and young children. It's more common in girls than boys. The condition can range from mild to severe and involves the hip socket not fully covering the ball portion of the upper thigh bone. This misalignment can result in the joint being loose and, in severe cases, it may dislocate.

There are several potential causes and risk factors for hip dysplasia, including:

1. **Genetic Factors**: A family history of hip dysplasia can increase the likelihood of the condition.

2. **Position in the Womb**: Babies who are in a breech position in the womb are more at risk.

3. **Birth Factors**: Firstborn children and those born with a low birth weight or after a difficult labor may have a higher risk.

4. **Lifestyle/Cultural Practices**: Certain swaddling or carrying practices that keep a baby's legs straight and together may contribute to the development of hip dysplasia.

Symptoms can include uneven leg lengths, uneven skin folds on the thighs, and a limited range of motion in one leg. In older children and adults, symptoms may involve hip pain and a limp.

Treatment depends on the age at diagnosis and the severity of the condition. It may involve the use of a brace or harness in infants to position the hip correctly as it develops. In more severe cases, or if diagnosed later in childhood, surgery might be necessary to correct the joint.

Early diagnosis and appropriate treatment are crucial to avoid long-term problems such as hip pain, uneven leg lengths, and early-onset arthritis. Regular pediatric check-ups often include screening for hip dysplasia.

1. **How is hip dysplasia diagnosed?**

Hip dysplasia is typically diagnosed through a combination of physical examinations and imaging tests. The approach to diagnosis can vary depending on the age of the patient:

1. **Newborns and Infants**:

- **Physical Examination**: In newborns, doctors often perform specific maneuvers during a physical exam to check for hip stability. These include the Ortolani Test and the Barlow Maneuver, which involve gently manipulating the legs and hips to check for any signs of dislocation or looseness in the hip joint.

- **Ultrasound**: For infants up to six months old, an ultrasound is often used. It's an effective tool because it can visualize the soft cartilage of a baby's hip joint, which is not yet visible on X-rays.

2. **Older Infants and Children**:

- **X-rays**: For older infants and children whose bones have started to ossify (harden), X-rays are typically used. They can show whether the ball and socket of the hip joint are properly aligned.

- **Physical Signs**: In older children, signs such as a limp, leg length discrepancy, or limited range of motion can prompt a doctor to order imaging tests.

3. **Adolescents and Adults**:

- **X-rays and MRI**: In older patients, X-rays are the primary diagnostic tool, but an MRI can also be used to get a detailed image of the hip joint, including soft tissues.

Other factors that doctors may consider during diagnosis include the patient's family history, any risk factors present (such as being in the breech position before birth), and the developmental history of the child. Early detection is key in managing hip dysplasia effectively, as it can prevent more severe complications later in life. Regular pediatric check-ups, especially during the first year of life, are crucial for early detection and treatment.

1. **What are symptoms of hip dysplasia (DDH)?**

Symptoms of Developmental Dysplasia of the Hip (DDH) can vary depending on the age of the individual and the severity of the condition. Here are some common symptoms:

1. **In Infants**:

- **Asymmetry in Thigh Folds**: Uneven skin folds on the thighs or buttocks.

- **Limited Range of Motion**: Difficulty spreading the legs apart during diaper changes.

- **Leg Length Discrepancy**: One leg may appear shorter than the other.

- **Clicking Sound**: A clicking or popping noise may be heard or felt during movement of the hip joint.

2. **In Older Children**:

- **Limping**: A child who is walking may limp or exhibit an abnormal gait.

- **Walking on Toes**: Walking on the toes of one foot, or a waddling walk.

- **Pain**: There may be discomfort or pain in the hip, especially during or after walking.

- **Leg Length Discrepancy**: Continuation or worsening of any length difference between legs.

- **Reduced Mobility**: Difficulty with movements that involve spreading the legs.

3. **In Adolescents and Adults**:

- **Hip Pain**: Chronic hip pain, often aggravated by activity.

- **Limping or Altered Gait**: Difficulty walking normally.

- **Osteoarthritis**: Early development of osteoarthritis in the hip joint.

- **Stiffness**: A feeling of stiffness or restricted movement in the hip.

It's important to note that in some cases, especially in infants, DDH may not present any noticeable symptoms. This is why routine pediatric check-ups are crucial, as healthcare providers will perform specific physical exams to screen for DDH. Early detection and treatment can significantly improve outcomes and reduce the risk of long-term complications.

1. **What happens to the hip in hip dysplasia?**

In hip dysplasia, the hip joint doesn't develop properly, leading to a mismatch between the ball (the femoral head at the top of the thigh bone) and the socket (the acetabulum in the pelvis). The main issues in hip dysplasia include:

1. **Shallow Acetabulum**: Normally, the acetabulum is a deep cup that covers the femoral head, providing stability and allowing smooth movement. In hip dysplasia, the acetabulum is too shallow, which means it doesn't cover the femoral head as completely as it should. This lack of coverage can lead to instability in the hip joint.

2. **Unstable or Loose Joint**: The shallowness of the socket and the improper alignment can cause the hip joint to be loose or unstable. In severe cases, this instability can lead to dislocation, where the femoral head slips out of the socket.

3. **Altered Joint Mechanics**: Because the joint doesn't fit together correctly, the mechanics of the hip are altered. This can lead to abnormal wear and tear on the joint surfaces and the labrum (the ring of cartilage around the socket).

4. **Increased Risk of Osteoarthritis**: Over time, the abnormal contact between the femoral head and the acetabulum can lead to premature wear of the joint, increasing the risk of developing osteoarthritis at a younger age than usual.

5. **Changes in Leg Length and Muscle Development**: In some cases, hip dysplasia can lead to differences in leg lengths and can affect the development of the muscles around the hip and leg.

The impact of hip dysplasia can vary greatly. Some individuals may have very mild forms and experience minimal symptoms, while others may have significant joint instability and discomfort, potentially requiring medical intervention such as bracing in infants or surgery in more severe cases or if diagnosed later in life. Early detection and treatment are crucial to improve the outcome and reduce the risk of long-term complications like chronic pain and mobility issues.

1. **What are treatment options?**

Treatment options for hip dysplasia, also known as Developmental Dysplasia of the Hip (DDH), vary depending on the age of the patient and the severity of the condition:

1. **Infants (Newborn to 6 Months)**:

- **Pavlik Harness**: This is often the first line of treatment. The harness is used to hold the baby's hip in the correct position, allowing for proper development of the joint.

- **Braces or Splints**: If the Pavlik harness is not effective or suitable, other types of braces or splints may be used.

2. **Infants and Children (6 Months to 18 Months)**:

- **Closed Reduction and Spica Cast**: If the hip is dislocated, a procedure called closed reduction may be performed under anesthesia to place the femoral head back into the socket, followed by immobilization with a spica cast.

- **Open Reduction**: If closed reduction is not successful or possible, an open surgical procedure may be needed to correctly position the hip.

3. **Older Children (Over 18 Months)**:

- **Surgical Intervention**: For older children, surgery is often required to correct the hip joint. This may involve cutting and repositioning the bones (osteotomy) and/or reconstructing the soft tissues around the hip.

- **Physical Therapy**: Post-surgical rehabilitation with physical therapy is important to improve strength and mobility.

4. **Adolescents and Adults**:

- **Osteotomy**: Surgical realignment of the hip bones can be performed to improve the fit and function of the hip joint.

- **Hip Replacement**: In cases where there is significant joint damage or arthritis, a total hip replacement may be considered.

5. **Non-Surgical Options**:

- **Observation and Monitoring**: In mild cases, especially in very young infants, the condition might improve on its own. Regular monitoring is necessary to ensure proper hip development.

- **Physical Therapy**: Physical therapy may be recommended to strengthen the muscles around the hip and improve range of motion.

- **Pain Management**: For older children and adults, managing pain and maintaining hip function are important aspects of treatment.

The choice of treatment depends on several factors, including the patient's age, the severity of the dysplasia, and whether the hip is dislocated. The goal of treatment is to achieve a stable and well-aligned hip joint to prevent long-term complications such as pain and osteoarthritis. Early diagnosis and intervention can significantly improve the outcome for individuals with hip dysplasia.

1. **What is done in periacetabular osteotomy?**

Periacetabular osteotomy, often referred to as PAO, is a surgical procedure used to treat hip dysplasia in adolescents and adults, particularly when conservative treatments are not effective. The primary goal of this surgery is to improve the alignment and function of the hip joint, thereby relieving pain and reducing the risk of early-onset osteoarthritis. Here's what typically happens during a PAO:

1. **Preoperative Assessment**: Before the surgery, detailed imaging (like X-rays and sometimes MRI) is done to plan the procedure and determine the specific changes needed in the hip's bone structure.

2. **Anesthesia**: The surgery is performed under general anesthesia, so the patient is asleep and does not feel pain during the operation.

3. **Surgical Procedure**:

- **Incision**: The surgeon makes an incision near the hip to access the pelvis and hip joint.

- **Cutting the Bone (Osteotomy)**: The surgeon performs carefully planned cuts (osteotomies) around the acetabulum – the socket part of the hip joint. These cuts are made in such a way that the socket can be repositioned without detaching it completely from the pelvis.

- **Repositioning the Socket**: The acetabulum is then rotated to a position where it covers the femoral head (ball part) more effectively. This reorientation improves the stability and mechanics of the hip joint.

- **Securing the Position**: Metal screws and plates are typically used to secure the repositioned acetabulum in its new place until the bones heal.

4. **Recovery and Rehabilitation**:

- **Hospital Stay**: The patient may stay in the hospital for a few days after surgery.

- **Pain Management**: Postoperative pain is managed through medications.

- **Physical Therapy**: Rehabilitation starts soon after surgery, focusing on regaining mobility and strength. It plays a crucial role in the recovery process.

- **Weight Bearing**: Initially, there might be restrictions on weight-bearing on the operated hip. Gradually, the patient can start putting weight on the hip as healing progresses.

5. **Follow-Up**:

- **Monitoring Healing**: Regular follow-up appointments are necessary to monitor the healing of the bone and the position of the hardware.

- **Long-Term Outcomes**: The long-term goal of PAO is to provide a more normally functioning hip joint, reduce pain, and delay or prevent the onset of osteoarthritis.

Periacetabular osteotomy is a complex procedure and is usually recommended when less invasive treatments are not suitable or have failed to relieve symptoms. It's important for patients undergoing PAO to have a clear understanding of the recovery process and to be committed to the necessary physical therapy for the best outcomes.

1. **Do I need to stop smoking before PAO?**

Yes, it is highly recommended to stop smoking before undergoing a Periacetabular Osteotomy (PAO) or any major surgical procedure. Smoking can significantly affect your surgery and recovery in several ways:

1. **Impaired Blood Flow and Healing**: Nicotine causes blood vessels to constrict, reducing blood flow. This can impair the healing process, as adequate blood flow is crucial for the delivery of oxygen and nutrients to the surgical site.

2. **Increased Risk of Infection**: Smoking weakens the immune system, increasing the risk of post-surgical infections.

3. **Poor Bone Healing**: Smoking has been shown to have a detrimental effect on bone healing. Since PAO involves cutting and repositioning bone, smoking can impede this crucial healing process.

4. **Increased Anesthetic Risk**: Smoking affects lung function, which can complicate the administration of anesthesia and increase the risk of respiratory complications during and after surgery.

5. **Delayed Recovery**: Smokers often have a longer recovery period and may experience more complications compared to non-smokers.

Given these risks, surgeons often advise patients to quit smoking well in advance of the surgery (typically several weeks to months before). This not only improves the surgical outcomes but also contributes to overall better health. Moreover, some surgeons may even require patients to quit smoking as a condition for performing elective surgeries like PAO.

It's advisable to discuss your smoking habits with your healthcare provider, who can provide guidance and resources to help you quit smoking before your surgery. Quitting smoking can be challenging, but doing so will significantly improve your chances of a successful surgery and a smoother recovery.

1. **What is the postoperative mobilization regimen? When can i walk after PAO?**

The postoperative mobilization regimen after a Periacetabular Osteotomy (PAO) is a critical aspect of recovery and varies based on individual patient factors, the specifics of the surgery, and the surgeon's protocol. Generally, the regimen is designed to balance the need for the bone to heal with the need to maintain muscle strength and joint mobility. Here's a typical timeline and approach to mobilization after PAO:

1. **Immediate Postoperative Period (First Few Days)**:

- Patients are typically encouraged to start moving as soon as possible to reduce the risk of blood clots and improve circulation. This often includes bed exercises like ankle pumps and gentle leg lifts.

- Walking may not be possible immediately, and mobility is usually assisted with the use of crutches or a walker.

2. **Early Recovery (First Few Weeks)**:

- Weight-bearing status is determined by the surgeon and can range from non-weight-bearing to partial weight-bearing. This depends on factors like the stability of the osteotomy and individual healing response.

- Physical therapy usually begins soon after surgery. Initially, it focuses on gentle exercises to improve hip motion and maintain muscle tone without stressing the healing bone.

3. **Gradual Weight-Bearing (4-6 Weeks Post-Surgery)**:

- Over time, as healing progresses, you may be allowed to gradually increase weight-bearing. This is often done in stages, starting with toe-touch or partial weight-bearing.

- Physical therapy will continue and will progressively include more strength and mobility exercises.

4. **Full Weight-Bearing (6-12 Weeks Post-Surgery)**:

- Most patients can expect to start full weight-bearing between 6 to 12 weeks after surgery, but this varies widely.

- The transition to full weight-bearing is gradual, and you will likely still need crutches or a walker initially.

5. **Ongoing Recovery and Rehabilitation (3-6 Months and Beyond)**:

- Complete healing and return to normal activities can take several months.

- Rehabilitation will continue to focus on strengthening and improving flexibility. The goal is to return to normal activities safely and effectively.

Throughout the recovery process, follow-up appointments with your surgeon are crucial. They will monitor your healing through physical exams and possibly imaging studies like X-rays. It's important to adhere to your surgeon's instructions regarding activity level and weight-bearing to ensure optimal healing.

Remember, every patient's recovery is unique, and timelines can vary. It's essential to communicate with your healthcare team and follow their guidance closely for the best outcomes.

1. **What are possible comlications in PAO?**

Periacetabular Osteotomy (PAO) is a complex surgical procedure, and like any major surgery, it carries certain risks and potential complications. It's important to be aware of these when considering or preparing for the surgery:

1. **Blood Loss**: Significant blood loss can occur during PAO, sometimes requiring transfusions.

2. **Infection**: As with any surgery, there is a risk of infection at the incision site or deeper, around the bone and implanted hardware.

3. **Nerve Damage**: The surgery involves operating near nerves; there's a risk of nerve damage, which could lead to numbness, tingling, or weakness in the legs.

4. **Blood Clots**: Being immobile after surgery increases the risk of developing blood clots in the legs (deep vein thrombosis) which could travel to the lungs (pulmonary embolism).

5. **Nonunion or Malunion of Bone**: There is a possibility that the bones may not heal properly (nonunion) or may heal in an incorrect position (malunion).

6. **Hip Stiffness or Reduced Range of Motion**: The hip joint may become stiff or have reduced mobility after surgery.

7. **Pain**: Some patients may continue to experience hip pain after the surgery, either due to the surgery itself or underlying issues not addressed by the PAO.

8. **Implant Problems**: Complications related to the screws and plates used to secure the hip, such as irritation or the need for removal, can occur.

9. **Leg Length Discrepancy**: The surgery may result in a slight difference in leg lengths.

10. **Osteoarthritis**: Although PAO aims to delay or prevent osteoarthritis, there's still a risk of developing arthritis in the hip over time.

11. **Need for Additional Surgery**: In some cases, further surgery may be required, either due to complications or because the initial surgery did not completely resolve the issue.

It's crucial to discuss these risks thoroughly with your orthopedic surgeon to understand their relevance to your specific situation. The surgeon will take steps to minimize these risks as much as possible. Additionally, your overall health, the severity of your hip dysplasia, and the skill and experience of your surgical team are significant factors in the likelihood and management of these complications. After the surgery, following your surgeon's and physical therapist's instructions closely can help minimize risks and aid in your recovery.

1. **When can I work after PAO?**

The timeline for returning to work after a Periacetabular Osteotomy (PAO) varies depending on several factors, including the nature of your job, your overall health, the specifics of your surgery, and your individual rate of recovery. Here's a general guide:

1. **Type of Work**:

- **Sedentary Jobs**: If your job is primarily desk-based with minimal physical activity, you might be able to return to work as early as 2-6 weeks post-surgery. This, of course, depends on your pain levels and ability to sit comfortably for extended periods.

- **Physically Demanding Jobs**: If your job involves manual labor, standing for long hours, or significant physical activity, you may need a longer period of recovery. Returning to such work might take several months.

2. **Recovery Progress**:

- Your ability to return to work depends heavily on your recovery progress, including pain management, mobility, and your ability to perform job-related tasks without risking injury.

3. **Surgeon’s Guidance**:

- Your surgeon will provide specific recommendations based on your postoperative condition and progress. It's important to follow these guidelines to avoid complications.

4. **Workplace Accommodations**:

- Discuss with your employer any potential temporary modifications or accommodations that could be made to ease your transition back to work. This might include options like working from home, part-time hours, or modified duties.

5. **Transportation Considerations**:

- If your commute involves driving, you'll need to be off certain pain medications and have sufficient mobility and reaction time to drive safely. This typically means waiting until you're no longer reliant on opioids for pain management and can comfortably operate the vehicle.

6. **Physical Therapy and Exercises**:

- Continuing with prescribed physical therapy and exercises is crucial during your return to work. This will help in regaining strength and function in your hip.

It's important to remember that every individual's recovery is different, and there's no one-size-fits-all timeline. Listening to your body and not rushing back to work before you're ready is key. Overexerting yourself too soon after surgery can delay healing and potentially lead to complications. Regular follow-up appointments with your healthcare provider will help assess your readiness to return to work.

1. **When can I play sports again after PAO?**

Returning to sports after a Periacetabular Osteotomy (PAO) depends on various factors including the type of sports, your individual recovery progress, the guidance of your healthcare team, and the specifics of your surgery. Here's a general overview of what to expect:

1. **Initial Recovery Phase (First 6-12 Weeks)**:

- During the early weeks post-surgery, high-impact activities and sports are generally avoided to allow proper healing of the bone and soft tissues.

- Focus is on basic mobility, strength, and range of motion exercises under the guidance of a physical therapist.

2. **Gradual Return to Activity (3-6 Months)**:

- As you heal and regain strength and mobility, you may gradually start low-impact activities such as swimming or cycling.

- High-impact, pivoting, or contact sports should still be avoided during this time.

3. **Assessment for Return to Sports (6-12 Months)**:

- The timeline for returning to sports varies widely among individuals.

- Usually, around the 6-month mark or later, your surgeon or physical therapist will assess your readiness to participate in more demanding physical activities.

- This decision is based on factors like the stability and strength of your hip, your overall physical fitness, and the type of sports you wish to return to.

4. **Guided Return to Sports**:

- When you're cleared to resume sports, it's typically recommended to start slowly and gradually increase intensity.

- You may need to modify your technique or the intensity of your play to accommodate your hip's new condition.

5. **Monitoring and Adjustment**:

- It's crucial to monitor for any pain, discomfort, or other signs that might indicate overexertion.

- Regular follow-ups with your healthcare team are important to ensure that your return to sports is not negatively impacting your hip.

6. **Long-Term Considerations**:

- Some high-impact or contact sports might carry a higher risk of injury or wear on the hip joint even after recovery. Discussing long-term risks and benefits with your healthcare provider is important.

Remember, this timeline can vary significantly based on individual circumstances. The key to a successful return to sports is to progress gradually and listen to your body, avoiding pushing yourself too hard too soon. Maintaining open communication with your healthcare team throughout this process is essential. They can provide tailored advice based on your recovery and athletic goals.

1. **How long will I take to heal after PAO?**

The healing time after a Periacetabular Osteotomy (PAO) can vary significantly depending on several factors, including the individual's overall health, the specifics of their surgery, their adherence to postoperative instructions, and their body's natural healing process. Here's a general timeline to give you an idea:

1. **Initial Healing Phase (First 6-12 Weeks)**:

- The first few weeks are crucial for the healing of the bone and soft tissues. This period typically involves limited weight-bearing, with gradual increases as advised by your surgeon.

- Pain and swelling are common, and managing these is a key part of early recovery.

2. **Intermediate Phase (3-6 Months)**:

- By this time, the bones are usually well on their way to healing, but it's still a period of strengthening and rehabilitation.

- Physical therapy is important during this phase to regain strength, mobility, and function in the hip.

3. **Full Recovery (6-12 Months and Beyond)**:

- Most patients can expect to see significant improvements by 6 months, but it can take up to a year or more for a full recovery.

- By one year, most of the healing should be complete, although some subtle improvements and adaptations can continue beyond this point.

4. **Return to Normal Activities**:

- The timeline for returning to normal activities, including sports and more strenuous tasks, varies. It often depends on how well you've regained strength and mobility in your hip.

- It's important to follow your surgeon's guidelines and not rush this process.

5. **Long-term Outcomes**:

- PAO aims to provide long-term relief from hip pain and improve joint function, potentially preventing or delaying arthritis.

- Ongoing care, including maintaining a healthy weight, regular exercise, and periodic check-ups, can be important for maintaining hip health.

Remember, these timelines are approximate and can vary widely among individuals. It's crucial to maintain open communication with your healthcare team throughout your recovery process. They can provide the best guidance tailored to your specific situation. Listening to your body and not pushing yourself too hard is key; every person's body heals at its own pace.

1. **How long does the surgery take?**

The duration of a Periacetabular Osteotomy (PAO) surgery can vary depending on several factors, including the complexity of the case, the specific techniques used by the surgeon, and whether any additional procedures are needed during the surgery. However, on average, PAO surgery typically takes about 2 to 4 hours.

This time frame includes:

1. **Preparation**: Getting the patient anesthetized and positioned properly for surgery.

2. **Surgical Procedure**: The actual time taken to perform the osteotomies (bone cuts), reposition the acetabulum (hip socket), and secure it with screws or other fixation devices.

3. **Closure**: Closing the surgical incision and ensuring hemostasis (control of bleeding).

It's important to note that this is just the time spent in the operating room. Additional time is needed for pre-operative preparations and post-operative recovery in the hospital. Also, the duration of the surgery does not directly correlate with the success or complexity of the procedure; each case is unique and requires individualized attention from the surgical team.

Your surgeon can provide a more specific estimate based on your individual circumstances and the surgical approach they plan to use.

1. **How long is the hospital stay after PAO?**

The length of hospital stay after a Periacetabular Osteotomy (PAO) can vary depending on several factors, including the specifics of the surgery, the patient's overall health, the rate of initial recovery, and the presence of any complications. On average, patients can expect to stay in the hospital for about 2 to 5 days after the surgery.

Here's a breakdown of what typically influences the length of the hospital stay:

1. **Postoperative Monitoring**: Immediately after surgery, close monitoring is necessary to manage pain, monitor for any complications, and initiate early mobilization.

2. **Pain Management**: Adequate control of postoperative pain is essential before discharge. This often involves transitioning from intravenous to oral pain medications.

3. **Mobility**: Patients are usually encouraged to start moving as soon as possible after surgery. The ability to perform basic movements and tasks, such as getting in and out of bed, walking with assistance, and using the bathroom, is a key factor in determining readiness for discharge.

4. **Physical Therapy Assessment**: A physical therapist will typically assess and work with the patient, providing exercises and guidelines for mobility and strength.

5. **Medical Clearance**: The surgical team will assess the wound site, ensure there are no signs of infection, and check that the patient is medically stable.

6. **Patient Education**: Before discharge, patients receive instructions on wound care, signs of complications to watch for, medication management, follow-up appointments, and guidelines for activity and rehabilitation at home.

7. **Support System**: The availability of a support system at home can also influence the length of stay. Patients who have help at home may be discharged earlier.

It's important to follow the advice and recommendations of your healthcare team regarding discharge and postoperative care. Each patient's recovery is unique, and your medical team will ensure that you're ready to continue your recovery safely at home before discharging you from the hospital.

1. **Will i need to wear a brace after PAO?**

After a Periacetabular Osteotomy (PAO), whether or not you need to wear a brace depends on your surgeon's protocol and the specifics of your surgery. The use of a brace post-PAO is not universally standard and varies based on individual cases and surgeon preferences. Here are some considerations:

1. **Surgeon's Protocol**: Some surgeons may prescribe a brace as part of the postoperative regimen to help stabilize the hip, limit certain movements, and aid in the healing process. Others might not find it necessary.

2. **Purpose of the Brace**: If used, a brace can help to:

- Provide support to the operated hip.

- Limit the range of motion to protect the surgical site.

- Assist in proper healing by keeping the hip in an optimal position.

3. **Type of Surgery and Healing Process**: The decision to use a brace can also depend on factors like the extent of the surgery, how well the bones are expected to heal, and the stability of the hip joint after surgery.

4. **Patient-Specific Factors**: Individual factors, such as the patient's overall health, activity level, and history of compliance with medical instructions, can also influence the decision.

5. **Duration of Use**: If a brace is prescribed, your surgeon will provide guidance on how long it should be worn. This can vary from a few weeks to several months, depending on your recovery progress.

6. **Alternatives to Bracing**: In some cases, instead of or in addition to a brace, specific movement restrictions and physical therapy protocols are used to ensure safe healing.

It's important to follow your surgeon's recommendations regarding the use of a brace. They will make a decision based on what's best for your specific situation and recovery goals. If you have any concerns or questions about using a brace after your surgery, be sure to discuss them with your healthcare provider.

1. **Will I need to have my screw removed after PAO?**

The necessity of screw removal after a Periacetabular Osteotomy (PAO) varies from patient to patient. Here are some factors to consider:

1. **Routine Practice**: Some surgeons routinely plan for the removal of screws after a certain period, often once the bones have adequately healed. This might be around 12 to 18 months post-surgery, but the timing can vary.

2. **Patient Comfort and Symptoms**: In some cases, the screws can cause discomfort or irritation, especially in thinner patients or if the screws are prominent under the skin. If you experience pain, tenderness, or other symptoms related to the hardware, your surgeon may recommend removal.

3. **Individual Circumstances**: The decision to remove the screws can depend on various factors, including your age, activity level, and personal preference, as well as the surgeon's assessment of how well the hip has healed.

4. **Additional Surgeries**: If any further hip surgeries are required, the surgeon might remove the screws during the subsequent procedure.

5. **No Symptoms**: If the screws are not causing any issues and the hip joint is functioning well, some surgeons and patients opt to leave the hardware in place indefinitely.

6. **Surgical Considerations**: Screw removal is a relatively minor procedure compared to the initial PAO, but it still involves surgery with its inherent risks, like infection and anesthesia-related risks.

It's important to have a detailed discussion with your orthopedic surgeon about the pros and cons of screw removal in your specific case. They will provide guidance based on your individual situation, the healing progress of your hip, and any symptoms you might be experiencing. Regular follow-up appointments after PAO are crucial to monitor your recovery and address any concerns, including those related to hardware.

1. **Does the labrum also need to be repaired in PAO?**

In a Periacetabular Osteotomy (PAO), whether the labrum needs to be repaired depends on the specific circumstances of the patient's hip condition. The labrum is a ring of cartilage that surrounds the acetabulum (hip socket) and helps to stabilize the hip joint. Here are some considerations:

1. **Condition of the Labrum**: The need for labral repair during PAO is primarily determined by the condition of the labrum itself. If the labrum is torn or damaged, which can sometimes occur as a result of the hip dysplasia, it may need to be repaired to restore the stability and function of the hip joint.

2. **Diagnosis and Imaging**: Prior to the PAO, imaging studies such as MRI or arthroscopy might be used to assess the condition of the labrum. If these studies reveal significant labral damage, the surgeon may plan to repair the labrum during the PAO.

3. **Intraoperative Assessment**: In some cases, the decision to repair the labrum is made during the surgery. The surgeon can directly assess the labrum once the hip joint is exposed and decide whether a repair is necessary.

4. **Symptoms and Functionality**: Symptoms such as pain, clicking, or locking of the hip joint might indicate a labral tear. The decision to repair the labrum may also be influenced by these symptoms and the overall functionality of the hip.

5. **Approach to Surgery**: Some surgeons may prefer to address any labral issues in a staged approach, where the labrum is assessed and repaired in a separate procedure either before or after the PAO, especially if arthroscopic techniques are being used.

6. **Long-term Outcomes**: Repairing a damaged labrum during PAO can potentially improve the long-term outcomes of the surgery, reducing symptoms and improving joint function.

It's important to discuss with your orthopedic surgeon the specifics of your case and whether labral repair will be part of your PAO. The surgeon's decision will be based on a combination of diagnostic imaging, your symptoms, and their findings during the surgery.

1. **Will I need hip replacement after PAO?**

A Periacetabular Osteotomy (PAO) is a surgical procedure primarily aimed at delaying or preventing the need for a hip replacement, especially in younger patients with hip dysplasia. The goal of PAO is to correct the alignment of the hip joint to improve its function and reduce pain, thereby potentially preventing or slowing the progression of osteoarthritis. However, whether a hip replacement becomes necessary later in life depends on several factors:

1. **Success of PAO**: If PAO successfully realigns the hip joint and relieves symptoms, it can significantly delay or even eliminate the need for a hip replacement.

2. **Progression of Joint Degeneration**: Hip dysplasia can lead to osteoarthritis over time. The extent of degeneration at the time of PAO and how the joint fares post-surgery play a role in determining the future need for a hip replacement.

3. **Patient's Age and Activity Level**: Younger, more active patients who undergo PAO may have different long-term outcomes compared to older patients or those with a sedentary lifestyle.

4. **Post-PAO Complications**: If complications arise after PAO, such as persistent pain, joint stiffness, or improper healing, these could potentially lead to the need for a hip replacement in the future.

5. **Long-term Hip Joint Health**: The overall health of the hip joint in the years following PAO, including how well arthritis is managed, can influence the eventual need for a hip replacement.

6. **Individual Variability**: Every patient is different, and so is their response to surgery and the progression of hip joint conditions.

It's important to have realistic expectations and understand that while PAO can be very effective in delaying the need for a hip replacement, it doesn't guarantee that a replacement will never be necessary. Regular follow-ups with your orthopedic surgeon and proper management of your hip health are crucial in monitoring the condition of your hip joint after PAO and in making future treatment decisions.

1. **When can I drive after PAO?**

The timeline for returning to driving after a Periacetabular Osteotomy (PAO) varies depending on several factors, including your overall recovery, the specific restrictions given by your surgeon, and which hip was operated on. Here are some general guidelines:

1. **Surgeon's Clearance**: Your surgeon will provide specific guidance based on your individual progress. It's crucial to get their clearance before you start driving.

2. **Pain and Medication**: You should not drive if you are taking narcotic pain medications, as these can impair your ability to drive safely.

3. **Strength and Mobility**: You need to have enough strength and mobility in your operated hip and leg to operate the pedals effectively and to move your foot from the gas to the brake pedal without hesitation.

4. **Right vs. Left Hip**:

- If your left hip was operated on and you drive an automatic transmission vehicle, you may be able to return to driving sooner than if your right hip was operated on, as the right leg is used for both gas and brake pedals.

- For manual transmission vehicles or if your right hip was operated on, the timeline might be longer.

5. **General Recovery Timeline**: Many patients can start driving 4-6 weeks after surgery, but this can vary. Some may need more time, especially if the surgery was on the right hip.

6. **Safety and Comfort**: Your ability to sit comfortably in the car and react quickly in traffic is crucial. You should also be able to perform an emergency stop without hesitation or pain.

7. **Trial Run**: Before driving on public roads, it may be advisable to test your driving ability in a safe and controlled environment, like an empty parking lot.

Remember, returning to driving should not be rushed. It's important for your safety and the safety of others on the road. Be sure to discuss your specific situation with your healthcare provider to get personalized advice.

1. **Why do I have to take blood thinners after PAO?**

Taking blood thinners after a Periacetabular Osteotomy (PAO) or any major orthopedic surgery is primarily for the prevention of blood clots. Here are the key reasons:

1. **Reduced Mobility**: After major surgery like PAO, your mobility is often limited during the initial recovery phase. Reduced mobility can slow down blood flow in the legs, increasing the risk of blood clots.

2. **Risk of Deep Vein Thrombosis (DVT)**: One of the potential complications following orthopedic surgery is the development of deep vein thrombosis, which is a blood clot that forms in a deep vein, usually in the leg. DVT can be serious because a clot can dislodge and travel to the lungs, causing a pulmonary embolism, which is a life-threatening condition.

3. **Prophylactic Treatment**: Blood thinners (anticoagulants) are used as a prophylactic (preventive) measure to reduce the blood's ability to clot. This helps to lower the risk of developing DVT during your recovery.

4. **Surgeon's Protocol**: The duration and type of blood thinner prescribed can vary based on your surgeon's protocol, your individual risk factors (such as personal or family history of blood clots), and the specifics of your surgery.

5. **Guidelines and Recommendations**: The use of blood thinners post-surgery is in line with medical guidelines for preventing postoperative venous thromboembolism (VTE), a term that includes both DVT and pulmonary embolism.

It's important to follow your surgeon's instructions regarding blood thinners, including how long to take them. Do not stop taking these medications without consulting your healthcare provider, as this could increase your risk of blood clots. If you have any concerns or experience potential side effects (such as unusual bleeding), you should contact your healthcare provider immediately.

1. **When can I have sex after PAO?**

Returning to sexual activity after a Periacetabular Osteotomy (PAO) is an important aspect of normal life and recovery, but it should be approached with care. The timeline can vary based on individual recovery, comfort levels, and the specifics of the surgery. Here are some general guidelines:

1. **Wait for Initial Healing**: Most surgeons recommend waiting until the initial postoperative pain has subsided and you can move comfortably. This is typically a few weeks after surgery, but it can vary.

2. **Surgeon's Clearance**: It's important to get clearance from your surgeon before resuming sexual activity. They can provide guidance based on your specific recovery progress.

3. **Pain and Comfort**: You should be free from significant pain and feel comfortable during the activity. If any position causes pain or discomfort, it should be avoided.

4. **Be Cautious with Hip Movements**: After PAO, your hip will have limitations in terms of movement and load-bearing. It's important to avoid positions that put too much stress or strain on the operated hip.

5. **Communication with Partner**: Open communication with your partner about your comfort, any limitations, and the need to be gentle is essential.

6. **Start Slowly**: When you do resume sexual activity, start slowly and gently. Listen to your body and avoid any movements that feel uncomfortable or painful.

7. **Use Supportive Positions**: Some positions may be more comfortable and safer for your hip than others. For example, being on the bottom with your partner taking most of the weight may be advisable initially. Pillows can be used for support and comfort.

8. **Monitor for Any Problems**: If you experience increased pain, swelling, or any other concerning symptoms during or after sexual activity, consult your healthcare provider.

The key is to approach this aspect of your recovery with patience and caution, respecting your body's limits as it heals from the surgery. Your surgeon or a physical therapist might also provide specific recommendations tailored to your situation.

1. **How often do I need to see the doctor after PAO and will I get x-rays?**

After a Periacetabular Osteotomy (PAO), regular follow-up visits with your doctor are essential to monitor your recovery and ensure proper healing. The frequency of these visits and the use of X-rays can vary, but here’s a general guideline:

1. **Initial Follow-up**: The first postoperative visit is typically scheduled within a few weeks after surgery. This visit usually includes an assessment of your incision, pain levels, and overall well-being.

2. **Regular Check-ups**:

- **Early Recovery Phase (First 3 Months)**: You might have appointments every 2-4 weeks. These visits often include X-rays to monitor the healing of the osteotomy and the position of any screws or plates.

- **Intermediate Phase (3-6 Months)**: Visits may be spaced out a bit more, possibly every 1-2 months, depending on your progress.

- **Long-term Follow-up (After 6 Months)**: Once your surgeon is satisfied with your initial healing, follow-up visits may occur at longer intervals, such as every 6 months to a year.

3. **X-rays**:

- **Routine Monitoring**: X-rays are a standard part of follow-up care to assess the healing of the bones and the position of the hip joint.

- **Frequency**: You can expect to get X-rays at most of your follow-up visits in the first year after surgery. The frequency may decrease as you progress further in your recovery.

- **Long-term Assessment**: Periodic X-rays may be recommended in the long term to monitor for any changes or developments in the hip joint, especially if you experience new or worsening symptoms.

4. **Assessment of Function and Mobility**: During your visits, your doctor will also assess your hip function, range of motion, strength, and mobility. They may adjust your physical therapy regimen based on this assessment.

5. **Addressing Concerns**: These visits are an opportunity to discuss any concerns or issues you may be experiencing, such as pain, mobility issues, or problems with your surgical incisions.

6. **Postoperative Complications**: In case of any signs of complications (like infection, issues with the surgical hardware, or problems with bone healing), more frequent visits might be necessary.

7. **Long-term Care**: Even after you've fully recovered, periodic check-ups may be recommended to monitor the long-term health of your hip.

The specific schedule for follow-up visits and X-rays can vary based on individual factors and the surgeon's protocols. It's important to adhere to the schedule provided by your healthcare team and attend all follow-up appointments to ensure the best outcome from your surgery.
